# P-Selectin-Mediated Platelet Adhesion Promotes the Metastasis of Murine Melanoma Cells

**DOI:** 10.1371/journal.pone.0091320

**Published:** 2014-03-14

**Authors:** Cui-Ling Qi, Bo Wei, Jie Ye, Yang Yang, Bin Li, Qian-Qian Zhang, Jiang-Chao Li, Xiao-Dong He, Tian Lan, Li-Jing Wang

**Affiliations:** 1 Vascular Biology Research Institute, Guangdong Pharmaceutical University, Guangzhou, China; 2 Department of Gastrointestinal Surgery, the Third Affiliated Hospital of Sun Yat-sen University, Guangzhou, China; University of Tennessee, United States of America

## Abstract

Studies have indicated that the aggregation of activated platelets with cancer cells facilitates tumor metastasis; the adhesion molecule P-selectin may be an important mediator of this process, but the detailed mechanism is unclear. In the current study, we established a B16F10 (B16) cell metastatic model in P-selectin knockout (P-sel^−/−^) mice to determine the effect of P-selectin-mediated platelet adhesion on metastasis. Compared with C57 mice, P-sel^−/−^ mice developed fewer metastatic foci, and cell proliferation within the metastatic tumors was inhibited by P-selectin deficiency. The platelet refusion assay demonstrated that mice with P-sel^−/−^ platelets developed fewer lung metastatic foci (P<0.01) with a lower microvascular density (MVD) than mice with wild-type platelets. A co-culture model of platelets and B16 cells was utilized to determine the difference in VEGF concentration in the supernatants. The results demonstrated that the supernatant from the P-sel^−/−^ platelet/B16 co-culture had a lower concentration of VEGF. Therefore, our findings indicated that P-selectin deficiency inhibited the metastasis of B16 cells and that wild-type platelet refusion reversed this inhibition. The P-selectin-mediated interaction between platelets and B16 cells promoted angiogenesis by up-regulating VEGF.

## Introduction

Cutaneous melanin pigment, which is synthesized from L-tyrosine and L-DOPA and excreted by melanocytes, plays an important role in camouflage and protection against solar radiation [1]. In response to multiple agents, melanocytes can become cancerous, resulting in the development of malignant melanoma. And also, melanogenesis can affect the behavior of melanoma cells [2]. In recent years, melanoma has become increasingly common, and its associated mortality rate is high. The prognosis for melanoma patients with distant metastases is especially poor, with a median survival time of only 8 months [3]. Melanoma cells tend to metastasize during early stages of tumorigenesis, largely because of their highly metastatic nature. Once metastasis occurs, the tumor becomes resistant to most therapies, including chemotherapy, radiotherapy, and immunotherapy [4].

Metastasis, which contributes to cancer-related death, requires a cascade of cellular events by which cancer cells establish new colonies at distant sites in the body [5]. Metastasis comprises multiple consecutive steps, such as local invasion, intravasation, migration in the circulatory system, extravasation, and establishment and growth in the target organ. Numerous cell adhesion molecules are involved in several stages of cancer metastasis [6], [7].

More than half a century ago, clinical observations and animal experiments indicated that tumor growth and metastasis could be inhibited by decreasing the number of platelets in the host blood [8], [9]. Subsequent studies found that platelets contribute to tumor metastasis by releasing chemokines and cytokines (such as VEGF and PDGF) and presenting several adhesion molecules, such as integrins and glycoproteins [10]. Recent data have indicated that P-selectin is a primary mediator [11]. P-selectin, which is expressed on stimulated endothelial cells and activated platelets, plays an important role in the binding of platelets to tumor cells and the adhesion of tumor cells to vascular endothelial cells [12], [13]. These particular processes are obligatory steps during tumor metastasis. The P-selectin-mediated aggregation of activated platelets with cancer cells can facilitate immune escape in the blood circulation. Furthermore, P-selectin-mediated adhesion of cancer cells to vascular endothelial cells contributes to microvascular arrest and the extravasation of cancer cells.

Given that decreasing platelets is not a feasible clinical approach because thrombocytopenia can cause hemorrhaging, a novel method of targeting the platelet-tumor cell interaction may produce a potential anti-metastatic treatment. Specifically, P-selectin may be a reasonable candidate. The current study aimed to determine the effect of P-selectin-mediated platelet adhesion on tumor metastasis and to provide a rationale for P-selectin inhibition as an anti-metastatic treatment.

## Materials and Methods

### Ethics Statement

All the animal experiments were performed according to the China National Guide for the Care and Use of Laboratory Animals and other relevant international guidelines. The current project was approved by the Medical Research Animal Ethics Committee of Guangdong Pharmaceutical University. Each researcher had the utmost concern for animal welfare during the entire study.

### Cell Culture

The mouse malignant melanoma B16F10 cell line (B16, ATCC, CCL-247) was obtained from Jian-Guo Geng′s laboratory at University of Michigan and was maintained in DMEM supplemented with 10% FBS, 200 µg/ml streptomycin and 200 U/ml penicillin at 37°C in a humidified 5% CO_2_/95% air atmosphere.

### Mice

P-selectin knockout mice (P-sel^−/−^ mice, 002289, with P-sel^−/−^ platelets) were obtained from The Jackson Laboratory (Maine, USA). The genetic identification of P-sel^−/−^ mice was made possible by the P-selectin gene and an extrinsic Neo gene. The two genes were detected by PCR using the following primers: P-selectin FW, 5′-TTGTAAATCAGAAGGAAGTGG-3′; P-selectin RV, 5′-AGAGTTACTCTTG ATGTAGATCTCC-3′; Neo FW, 5′-CTGAATGAACTGCAGGACGA-3′; and Neo RV, 5′-ATACTTTCT CGGCAGGAGCA-3′. Female mice at 6–8 weeks of age were included in the experimental groups. C57 mice (SCXK2008-0002, control with wild-type platelets) were purchased from Guangdong Medical Animal Experiment Center (Guangdong, China). All the mice were fed a ^60^Coγ-irradiated bacteria-free diet and were raised in specific pathogen-free (SPF) grade M5-type rearing cages under constant humidity and temperature.

### In vivo Metastasis Model

The metastasis model was established to determine the effect of P-selectin-mediated platelet adhesion on tumor metastasis. Logarithmic phase B16 cells were digested with 2.5 g/L trypsin and resuspended in serum-free DMEM. The percentage of living cells was determined by trypan blue staining, and at least 95% living cells were required. The B16 cells (1×10^5^ cells in 0.2 ml per mouse) were intravenously injected into P-sel^−/−^ (n = 16) and C57 (n = 25) mice. The mice were maintained as before, and the overall health condition was recorded in detail once a day. After 20 days, the mice were anesthetized with 10 mg/kg ketamine, 1 mg/kg atropine and 20 mg/kg xylazine hydrochloride by peritoneal injection and then sacrificed by cervical dislocation. Organ metastases were observed by gross anatomy. HE staining of the metastatic tumor tissue sections was performed to confirm homology with B16 cells. In each group, the number of metastatic foci (pigmented foci at the surface of the organ) was counted, and pictures were taken of the organs containing metastases. The metastatic area percentage was calculated using Image-Pro Plus 6.0 software according to the following formula: metastatic area percentage = pigmented area/total surface area. The number of metastatic and micrometastatic foci in the bulk tissue of a metastasis-containing organ was calculated with a low power lens. Fontana-Masson melanin staining of B16 cells and lung metastatic foci was performed to validate the quality of pigmentation in the injected cells and the secondary tumors.

### BrdU Assay

A BrdU cell proliferation assay was performed to determine whether the cell proliferation within metastatic tumors was influenced by P-selectin deficiency. BrdU (Sigma-Aldrich Corp) was administered intraperitoneally into the aforementioned metastasis model at a dose of 50 mg/kg; after 1 h, the P-sel^−/−^ mice were sacrificed. Three-micrometer paraffin sections of the lung and liver metastases were prepared for specific immunostaining with BrdU. The deparaffinized and hydrated sections were incubated in a 3% H_2_O_2_-methanol solution at 37°C for 30 min. Next, the washed sections were reconditioned with 0.4% pepsin for 30 min at 37°C. After blocking with 10% BSA for 1 h, the sections were incubated with a mouse anti-BrdU antibody (Santa Cruz Biotechnology) at 4°C overnight. Dimethylaminoazobenzene (DAB) coloration was performed after an incubation with an avidin/biotinylated horseradish peroxidase-labeled goat anti-mouse IgG (Santa Cruz Biotechnology). After counterstaining with hematoxylin, the mean number of BrdU-positive cells per random high power field (×400) was determined in each mouse using a light microscope.

### Platelet Refusion Assay

Anticoagulated blood from P-sel^−/−^ and C57 mice obtained by cardiac puncture under general anesthesia was centrifuged at 280 g for 8 min or 280 g for 4 min, respectively. Platelet-rich plasma (PRP) was harvested from the supernatants. Platelets were isolated by filtering the resulting PRP through a Sepharose 2B column (Sigma) equilibrated with Pipes Buffer. Eighteen P-sel^−/−^ mice were randomly divided into the experimental (n = 10) and control (n = 8) groups. Four hours after an intravenous injection of B16 cells (1×10^5^/mouse), each P-sel^−/−^ mouse received an intravenous injection of 4×10^7^ P-sel^−/−^ (experimental group) or wild-type (control group) murine platelets. After 20 days, the mice were sacrificed by cervical dislocation under anesthesia, and the liver and lung metastatic foci were counted by gross anatomy.

### Immunohistochemistry

Immunohistochemistry against CD31 was performed to determine the microvascular density (MVD) of the lung metastases and to investigate the influence of P-sel^−/−^ (experimental group) or wild-type (control group) platelet refusion on angiogenesis. A mouse anti-CD31 antibody (Santa Cruz Biotechnology) and an avidin/biotinylated horseradish peroxidase-labeled goat anti-mouse IgG (Santa Cruz Biotechnology) were used. Immunohistochemical staining was performed according to the protocol provided by the manufacturer. The mean number of CD31-positive vessels (denoted as the MVD) was determined based on a random low power field (×200) in each mouse using a light microscope. The data from each of these assays were collected using a double-blind protocol and were independently assessed by two experimenters (Cui-Ling Qi and Bo Wei).

### ELISA

Anticoagulated mouse blood collected from P-sel^−/−^ and C57 mice under general anesthesia was centrifuged at 400 g for 15 min, and the resulting supernatants were centrifuged for 15 min at 275 g or 1500 g, respectively. The harvested sediments were resuspended in DMEM. The platelets were activated with 10 µmol/L adenosine diphosphate (ADP) for 15 min at room temperature.

Logarithmic phase B16 cells were digested, resuspended, adjusted to a cell density of 1×10^5^/ml, seeded in 24-well plates at 1 ml/well, and routinely maintained in FBS-containing DMEM. When the cells adhered to the bottom, the culture solution was removed, and DMEM (blank control group) and activated platelets from P-sel^−/−^ (experimental group) or C57 (control group) mice were added. After culture at 37°C in a humidified 5% CO_2_/95% air atmosphere for 24 h, the supernatants were harvested for ELISA according to the protocol provided by the manufacturer (R&D Systems). The experiment was performed three times.

### Statistical Analysis

Statistical significance was determined by one-way ANOVA followed by the Bonferroni post-hoc test for multiple comparisons or Student’s t-test. For all tests, P<0.05 and P<0.01 were considered statistically significant and very significant, respectively.

## Results

### P-selectin Deficiency Inhibited the Metastasis of B16 Cells

**Figure 1:**
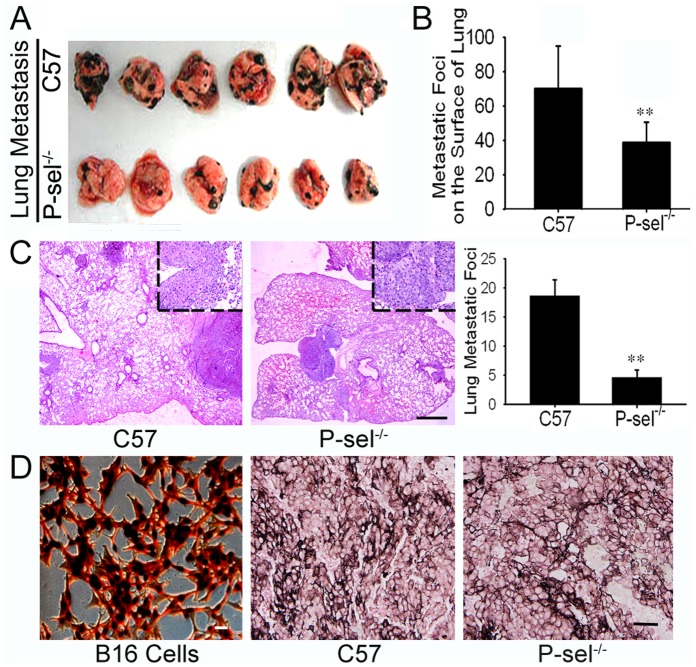
P-selectin deficiency inhibited B16 lung metastasis in P-sel^−/−^ mice. (A) Gross anatomy of B16 lung metastases in C57 and P-sel^−/−^ mice. (B) The quantification of the metastatic foci indicated that P-sel^−/−^ mice developed fewer metastatic foci on the lung surface than C57 mice. (C) HE staining of the metastatic tumors confirmed their homology with B16 cells, and the metastatic foci quantification in the bulk tissue indicated that P-sel^−/−^ mice developed fewer metastatic and micrometastatic foci than C57 mice. Scale bars: 100 µm. (D) Fontana-Masson melanin staining of B16 cells and lung metastases. Scale bars: 20 µm. **P<0.01.

**Figure 2:**
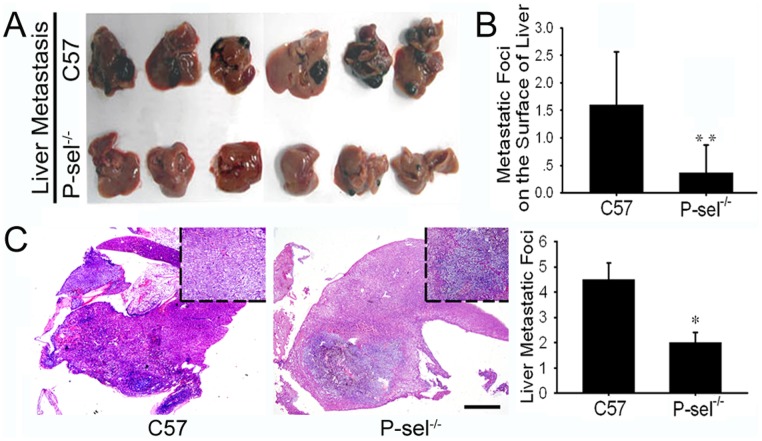
P-selectin deficiency inhibited B16 liver metastasis in P-sel^−/−^ mice. (A) Gross anatomy of the B16 liver metastases in C57 and P-sel^−/−^ mice. (B) The metastatic foci quantification indicated that P-sel^−/−^ mice developed fewer metastatic foci on the surface of the liver than C57 mice (P<0.01). (C) HE staining of bulk tissue revealed that P-sel^−/−^ mice developed fewer metastatic and micrometastatic foci than C57 mice (P<0.05). Scale bars: 100 µm. *P<0.05, **P<0.01.

**Figure 3:**
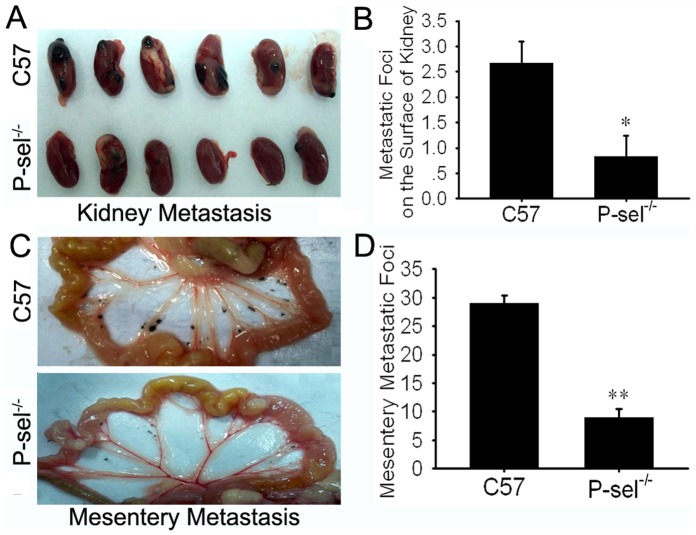
P-selectin deficiency inhibited B16 kidney and mesentery metastasis in P-sel^−/−^ mice. (A) Gross anatomy of B16 kidney metastases in C57 and P-sel^−/−^ mice. (B) Quantification of the metastatic foci indicated that P-sel^−/−^ mice developed fewer metastatic foci on the surface of the kidney than C57 mice (P<0.05). (C) Gross anatomy of the B16 mesentery metastases in C57 and P-sel^−/−^ mice. (D) The metastatic foci quantification demonstrated that P-sel^−/−^ mice developed fewer mesentery metastatic foci than C57 mice (P<0.01). *P<0.05, **P<0.01.

The genetic identification of the P-sel^−/−^ mice demonstrated homozygous deficiency of the P-selectin gene and expression of the extrinsic Neo gene (data not shown). Twenty days after intravenous injection of B16 cells, the mice were sacrificed, and organ metastasis was observed by gross anatomy. The metastatic foci exhibited melanin pigmentation, inequality of size at the organ surface. Lung metastases were found in all mice. Metastases were also detected in the liver, kidney and mesentery. HE staining of the metastases confirmed their homology with B16 cells. P-sel^−/−^ mice developed fewer metastatic foci on the surface of the above organs than C57 mice (figures 1A, 1B, 2A, 2B, and 3 and Table 1). The metastatic area percentages gave results similar to the enumeration of the metastatic foci (Table 1). The quantification of the metastatic and micrometastatic foci in the bulk lung and liver tissue under low power magnification also produced similar results (figures 1C and 2C). As for the metastasis in lymphatic system, we only found one metastatic foci in pulmonary hilar lymph node in each group (see in figure S1). Fontana-Masson staining showed a high density of melanin in B16 cells, and melanin positivity was observed in the lung metastases. There was no difference in pigmentation in the metastatic tumors in C57 and P-sel^−/−^ mice (figure 1D).

**Table 1:** Metastatic foci and metastatic area percentage for B16 cells in C57 and P-sel^−/−^ mice.

Metastatic Organ	C57	P-sel^−/−^	P value
Lung			
Metastasis foci	70.16±24.73	39.13±11.62	0.005
Metastatic area percentage (%)	5.21±0.76	1.62±0.41	0.002
Liver			
Metastasis foci	1.92±0.61	0.50±0.43	0.008
Metastatic area percentage (%)	2.98±0.35	0.21±0.13	<0.001
Kidney			
Metastasis foci	2.67±0.42	0.83±0.40	0.032
Metastatic area percentage (%)	2.33±0.83	0.26±0.14	0.035

### P-selectin Deficiency Inhibited Cell Proliferation in the Metastatic Tumors

**Figure 4:**
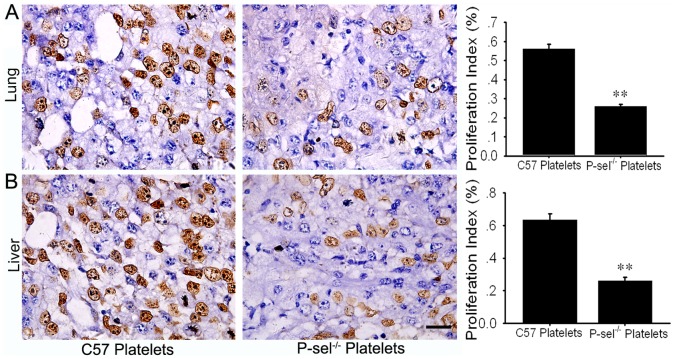
P-selectin deficiency inhibited cell proliferation within the metastatic tumors. (A) There were fewer BrdU-positive cells in the lung metastases in P-sel^−/−^ mice than in C57 mice (P<0.01). (B) There were fewer BrdU-positive cells in the liver metastases in P-sel^−/−^ mice than in C57 mice (P<0.01). Scale bars: 20 µm.

To determine the influence of P-selectin on cell proliferation within the metastatic tumors, BrdU cell proliferation assays were performed. The number of BrdU-positive cells in each high power field was calculated, and the statistical analysis indicated that there were fewer BrdU-positive cells in the lung and liver metastases in P-sel^−/−^ mice than in those from C57 mice (P<0.05; Figure 4).

### Wild-type Platelet Refusion Promoted the Metastasis of B16 Cells in P-sel^−/−^ Mice

**Figure 5:**
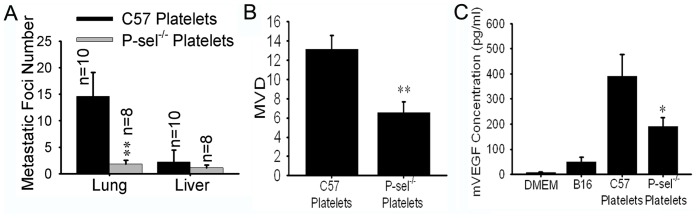
Wild-type platelet refusion promoted metastasis and angiogenesis by up-regulating VEGF. (A) Compared to mice with C57 platelets, mice in the experimental group with P-sel^−/−^ platelets developed fewer metastatic foci in the lungs (P<0.01). (B) Compared with the control group, the MVD was decreased in the experimental group (mice with P-sel^−/−^ platelets; P<0.01). (C) The supernatant from the wild-type platelet/B16 co-culture had a higher concentration of VEGF than the supernatant from the P-sel^−/−^ platelet/B16 co-culture.

To determine whether platelets expressing P-selectin promoted the metastasis of B16 cells in P-sel^−/−^ mice, the platelet refusion assay was performed. Most P-sel^−/−^ mice developed lung and liver metastases. Compared with the control group, mice in the experimental group with P-sel^−/−^ platelets developed fewer lung metastatic foci than those in the control group (P<0.01). Likewise, mice in the experimental group developed fewer liver metastatic foci than did those in the control group, but the difference was not statistically significant (P>0.05; Figure 5A).

### The P-selectin-mediated Interaction between Platelets and B16 Cells Promoted Angiogenesis by Up-regulating VEGF

The MVD of the lung metastases was assessed by immunochemistry against CD31. Compared with the control group, there was a lower MVD in the experimental group, which included mice with P-sel^−/−^ platelets (P<0.05; Figure 5B). To identify the reason for this difference, a co-culture model of platelets and B16 cells was utilized to determine the difference in VEGF concentration in the supernatants. The supernatant from the wild-type platelet/B16 co-culture had a higher concentration of VEGF (Figure 5C). Therefore, wild-type platelet refusion promoted VEGF production, thereby facilitating the vascularization of metastatic tumors. As a result, the P-sel^−/−^ mice with wild-type platelets (in the control group) developed more lung metastatic foci.

## Discussion

Hematogenous metastasis is a highly coordinated, sequential, dynamic, multistep process in which cancer cells interact with a variety of host cells. The cancer cells attain a mesenchymal phenotype via epithelial-mesenchymal transition (EMT), penetrate the basilar membrane, invade blood vessels and become circulating tumor cells (CTCs) [14], [15], [16]. Most of the CTCs are damaged during circulatory transport. This circulatory trauma may be caused by humoral and cellular immunity as well as by mechanical damage during passage through the microvasculature. The CTCs can be arrested in the capillary vessels because of mechanical microembolization or cell adhesion to endothelial cells; these cells subsequently extravasate out of the vessel and establish a secondary metastatic colony. Platelet involvement has long been recognized during the stages of circulatory transport and extravasation [8], [9], [17]. However, the mechanisms by which CTCs interact with host platelets and by which platelets promote tumor metastasis remain controversial. Our previous findings indicated that the adhesive molecule P-selectin is an important mediator between platelets and CTCs [18].

The selectin (CD62) family comprises a unique subfamily of Ca^2+^-dependent lectins that mediate adhesive interactions between platelets, leukocytes and vascular endothelial cells and that play an important role in inflammation, coagulation and wound healing [19]. P-selectin (CD62P) is an isoform in the selectin family, which mainly resides in α-granules of platelets and in the Weibel-Palade body of vascular endothelial cells. P-selectin is an ecto-lectin with an epidermal growth factor-like domain, a transmembrane domain and a cytoplasmic tail [18]. P-selectin is expressed on stimulated endothelial cells and activated platelets and plays an important role in the binding of platelets to tumor cells and the adhesion of tumor cells to vascular endothelial cells.

The current study aimed to validate the P-selectin-mediated platelet/CTC interaction that promotes the metastasis of mouse malignant melanoma B16–F10 cells using P-sel^−/−^ mice. Melanomas are highly malignant tumors, and melanoma cells tend to metastasize early in the tumorigenic process. Once metastasis occurs, the tumor becomes resistant to most therapies, leading to a very poor prognosis. Melanoma cells retain the function of melanogenesis, and pigmentation occurs at metastatic sites [2]. Recently, researchers found that melanogenesis decreases the overall survival and disease-free survival in patients with metastatic melanoma [20]. Our findings revealed melanogenesis in metastatic tumor tissue as in the inoculated B16 cells. The melanoma cells in the metastases retained the ability to perform melanogenesis. There was no difference in cellular melanogenesis in the metastatic tumors in C57 and P-sel^−/−^ mice.

A recent study demonstrated the involvement of platelets in the pulmonary metastasis of B16 cells [21]. Our findings indicated that P-sel^−/−^ mice developed fewer metastatic foci than C57 mice in the lung, liver, kidney and mesentery. P-selectin deficiency inhibited tumor metastasis. Via P-selectin, platelets aggregated with B16 cells in the circulatory system, and this type of tumor cell-induced platelet aggregation (TCIPA) is advantageous for the successful metastasis of B16 cells. B16 cells can escape immunolesion and physical damage when coated with platelets [8], [9], [22]. However, these tumor-platelet aggregates can become arrested in the microvasculature [23]. P-selectin also mediates the adhesion of B16 cells to vascular endothelium [24]. The BrdU cell proliferation assays demonstrated that P-selectin deficiency inhibited cell proliferation within the metastatic tumors; this may due to P-selectin-mediated platelet aggregation and the release of several growth factors [25], but this was not confirmed by ELISA (data not shown).

The above findings were confirmed using the platelet refusion assay. In the metastasis model, the P-sel^−/−^ mice with P-sel^−/−^ platelets developed fewer lung metastatic foci than those injected with C57 platelets (p<0.01). This indicated that P-selectin-mediated platelet adhesion promoted tumor metastasis. The CD31 immunochemistry results revealed a higher MVD in the metastatic tumors in the lungs of animals in the control group (P-sel^−/−^ mice with wild-type platelets) compared with those in the experimental group (p<0.05). Our study investigated the interaction between B16 cells and wild-type or P-sel^−/−^ platelets using a co-culture system. The ELISA results indicated that there was less VEGF in the supernatant from the P-sel^−/−^ platelet/B16 co-culture than in the supernatant from the C57 platelet co-culture. In conjunction with P-selectin, platelets promoted tumor-induced angiogenesis by releasing VEGF.

According to our data, P-selectin deficiency inhibited the metastasis of B16 cells, and the refusion of platelets expressing P-selectin reversed this inhibition. The interaction between the platelets and the B16 cells promoted the metastasis of B16 cells, and P-selectin was an important mediator of this interaction. The P-selectin-mediated interaction between platelets and B16 cells promoted angiogenesis by up-regulating VEGF, which is a potential mechanism by which platelet adhesion promotes metastasis. The results of the current study provide a rationale for P-selectin inhibition as an anti-metastatic treatment.

## Supporting Information

Figure S1 **Metastasis in lymph node.** Only one metastatic foci in pulmonary hilar lymph node in each group.
(TIF)
