# Supplementary figures and images for: P-Selectin-Mediated Platelet Adhesion Promotes the Metastasis of Murine Melanoma Cells
Source: PLoS One. 2014 Mar 14;9(3):e91320. doi: 10.1371/journal.pone.0091320 (PMC3954694; doi:10.1371/journal.pone.0091320)

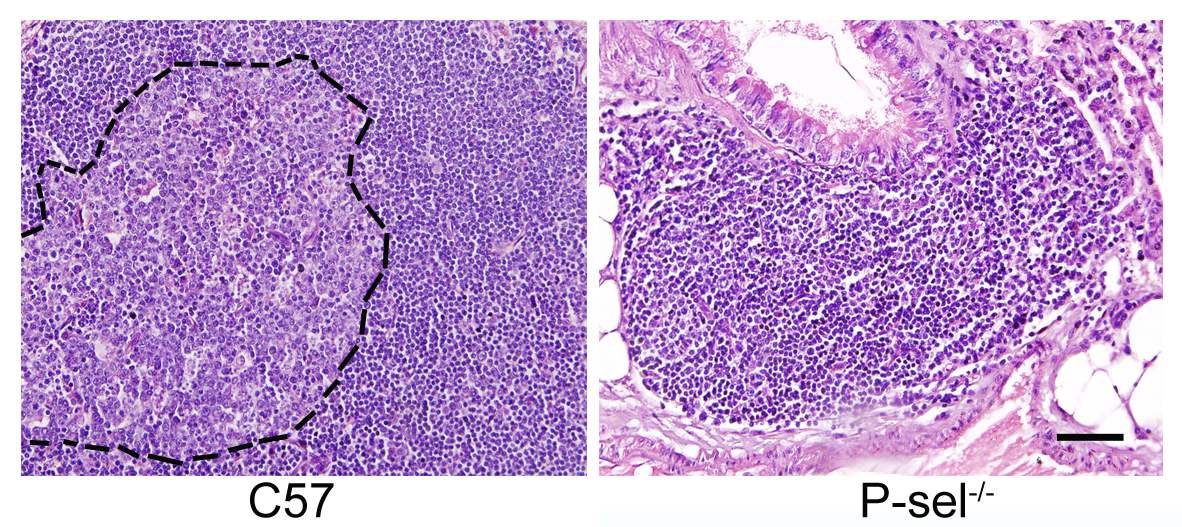

Supplement: Figure S1 — Metastasis in lymph node. Only one metastatic foci in pulmonary hilar lymph node in each group. (TIF) [file pone.0091320.s001.tif]
